# Idiopathic remitting seronegative symmetrical synovitis with pitting edema syndrome associated with bilateral pleural and pericardial effusions: a case report

**DOI:** 10.1186/s13256-016-0983-7

**Published:** 2016-07-20

**Authors:** Shozaburo Yanamoto, Jiro Fukae, Yurie Fukiyama, Shinsuke Fujioka, Shinji Ouma, Yoshio Tsuboi

**Affiliations:** Department of Neurology, Fukuoka University School of Medicine, 7-45-1 Nanakuma, Jonan-ku, Fukuoka, 814-0180 Japan

**Keywords:** RS3PE syndrome, Pleural effusions, Pericardial effusions, VEGF, IL-6

## Abstract

**Background:**

Remitting seronegative symmetrical synovitis with pitting edema syndrome is characterized by symmetrical synovitis with pitting edema in the dorsum of the hands or feet. Most cases of remitting seronegative symmetrical synovitis with pitting edema syndrome are idiopathic, but some are secondary to malignancy, autoimmune disease, or neurodegenerative disorders. Pleural and pericardial effusions are unusual complications in idiopathic remitting seronegative symmetrical synovitis with pitting edema syndrome.

**Case presentation:**

A 74-year-old Japanese woman presented to our hospital with arthralgia and pitting edema in her feet. She had pain in multiple joints, peripheral edema, and a markedly elevated erythrocyte sedimentation rate. Enhanced computed tomography and laboratory data showed no evidence of malignancy. These findings suggested that she had idiopathic remitting seronegative symmetrical synovitis with pitting edema syndrome. She also developed respiratory distress because of bilateral pleural and pericardial effusions. Laboratory data showed that serum vascular endothelial growth factor and interleukin-6 were significantly elevated. After administration of steroids, her pleural and pericardial effusions decreased and finally disappeared. Furthermore, vascular endothelial growth factor and interleukin-6 decreased when the pleural and pericardial effusions disappeared.

**Conclusions:**

Here we report the case of a patient with idiopathic remitting seronegative symmetrical synovitis with pitting edema syndrome associated with life-threatening complications, including bilateral pleural and pericardial effusions during the course of the illness, which led to respiratory failure and atrial fibrillation. Elevated vascular endothelial growth factor and interleukin-6 may be associated with the cause of pleural and pericardial effusions in idiopathic remitting seronegative symmetrical synovitis with pitting edema syndrome.

## Background

Remitting seronegative symmetrical synovitis with pitting edema (RS3PE) syndrome is characterized by symmetrical synovitis with pitting edema in the dorsum of the hands or feet, predominantly involving the hands [1–3]. These symptoms show a good response to low-dose corticosteroids, and remission can be maintained [1–3]. RS3PE syndrome shows a benign clinical course because of the good response to steroid treatment. However, patients with RS3PE syndrome rarely develop pleuritis, which induces pleural effusion [4–8]. Here we report the case of a patient with RS3PE syndrome associated with life-threatening complications including bilateral pleural and pericardial effusions during the course of the illness, which led to respiratory failure and atrial fibrillation.

## Case presentation

A 74-year-old Japanese woman gradually developed general malaise and loss of appetite. In addition, arthralgia and pitting edema in her feet appeared. Her laboratory findings revealed an elevated white blood cell (WBC) count and C-reactive protein (CRP). She was admitted to our hospital for further examination. She had an operation for endometrial cancer at the age of 72 and had no family history.

On admission, her blood pressure was 129/76 mmHg, heart rate was 101/minute, respiratory rate was 18/minute, and body temperature was 39.1 °C. A physical examination revealed pitting edema on the dorsum of her hands and feet. Her neurological examination was unremarkable. However, she had difficulty with squatting and walking because of arthralgia in her proximal lower limbs. Laboratory testing showed a WBC of 11700/μL, CRP of 6.7 mg/dL, erythrocyte sedimentation rate of 44 mm/hour, total protein of 5.8 g/dL, and albumin of 2.3 g/dL. Rheumatoid factor, anti Ro/SSA antibody, anti La/SSB antibody, anti Scleroderma 70 antibody, and anti cyclic citrullinated peptide antibody were negative. Serum interleukin-6 (IL-6) and vascular endothelial growth factor (VEGF) were markedly increased to 285 pg/mL (normal; <2 pg/mL) and 1820 pg/mL (normal; 262±228 pg/mL) [9], respectively. Her human leukocyte antigen (HLA) typing included B7. A chest X-ray revealed mild pleural effusion on the right side. Electrocardiography showed a complete right bundle branch block without ST-T change. Echocardiography revealed a slight pericardial effusion surrounding her entire heart.

**Fig. 1:**
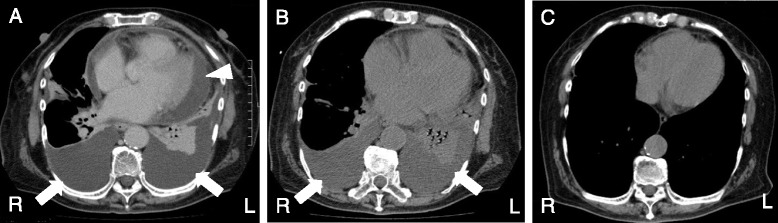
Chest computed tomography. **a** Chest computed tomography on the day before steroid therapy showed moderate bilateral pleural (*arrows*) and pericardial effusions (*arrowhead*). **b** Chest computed tomography after steroid therapy showed that the pleural effusion had decreased (*arrows*) and the pericardial effusion had disappeared. **c** Follow-up chest computed tomography on day 38 showed no pleural or pericardial effusions

She had a high fever, general malaise, and muscle weakness, and her laboratory tests revealed elevated inflammatory markers including WBC, CRP, and erythrocyte sedimentation rate. Because infectious disease was suspected, tazobactam-piperacillin 13.5 mg/day was started. On day 2, atrial fibrillation appeared on the electrocardiography monitor. A chest X-ray showed that the pleural effusion had increased in both lungs. Furthermore, echocardiography demonstrated increased pericardial effusion around her heart. On day 3, she developed respiratory failure, and oxygen administration was started by nasal cannula at a dose of 3 L/minute. Enhanced chest-abdominal computed tomography (CT) showed marked bilateral pleural and pericardial effusions without neoplastic lesions (Fig. 1a). Analysis of the pleural effusion revealed exudate with an increased protein level and neutrophils, but cytological evaluation of the pleural effusion revealed no evidence of malignancy. Cultures of blood and pleural effusion were all negative. Our patient fulfilled the diagnostic criteria of RS3PE syndrome including: 1) pitting edema of the hands and feet; 2) sudden onset of polyarthritis; 3) onset at age 50 years or older; and 4) negative serology for rheumatoid factors [1]. From day 6, she was treated with methylprednisolone administered intravenously at a dose of 1000 mg/day for 3 days. After steroid therapy, her fever rapidly improved, and blood tests revealed a decrease in CRP to 1.71 mg/dL. The pleural and pericardial effusions on chest CT decreased (Fig. 1b). When the pericardial effusion decreased, her atrial fibrillation disappeared. After steroid pulse therapy, oral prednisolone administration at a dose of 15 mg/day was started. Her respiratory failure immediately improved, and then she no longer required oxygen administration. A chest CT performed on day 38 revealed that the pleural and pericardial effusions had disappeared (Fig. 1c). On day 45, she was discharged. One year later, the prednisolone dosage was decreased to 8 mg/day, but the patient experienced no recurrence of symptoms. At the 1-year examination, no malignancy was found. Serum IL-6 and VEGF were significantly decreased to 1.3 pg/mL and 562 pg/mL, respectively.

## Discussion

RS3PE syndrome was first reported in 1985 by McCarty *et al*. and is characterized by: 1) good prognosis (Remitting); 2) negativity for antibodies, including rheumatoid factors (Seronegative); 3) symmetry (Symmetrical); and 4) synovitis with pitting edema of the dorsum of the hands and feet (Synovitis with Pitting Edema) [1]. Other common characteristics of RS3PE syndrome include acute onset in older persons, absence of bone erosion, inflammatory findings on blood examination, and painless limitation of movement of the wrist and fingers [2, 3]. Most cases of RS3PE syndrome are idiopathic, although the syndrome has been associated with malignancy, polymyalgia rheumatica, Parkinson’s disease, Sjögren’s syndrome, and ankylosing spondylitis [2, 3]. Our patient had acute onset of bilateral shoulder pain and edema on the dorsum of both feet at the age of 74. These symptoms were resolved by treatment with steroids, indicating the diagnosis of RS3PE syndrome. Because her enhanced CT and laboratory data showed no disease that may have induced RS3PE syndrome, she was diagnosed with idiopathic RS3PE syndrome.

Previously, five case reports of pleural effusion in patients with RS3PE syndrome have been reported (Table 1). Four cases showed the presence of pleural effusion in idiopathic RS3PE syndrome [4–7]. In these cases, the pleural effusion responded well to steroid therapy [4–7]. Taken together with our patient, pleuritis may occur rarely in patients with RS3PE syndrome. One case with RS3PE syndrome had both pleural and pericardial effusions [8]. However, this case of RS3PE syndrome was secondary to angioimmunoblastic T-cell lymphoma [8], which may induce pleural and pericardial effusions. Our case showed that pericarditis-induced pericardial effusion occurred in the patients with idiopathic RS3PE syndrome.

**Table 1:** Pleural and pericardial effusion associated with remitting seronegative symmetrical synovitis with pitting edema syndrome

Case	Age (years)	Sex	Pleural effusion	Pericardial effusion	ESR (mm/hour)	CRP (mg/dL)	Treatment	Prognosis	Ref.
1	88	M	Effusion	Negative	62	12.9	Half steroid pulse^a^ + PSL	Good	[4]
2	68	F	Exudative effusion (increased neutrophils)	Negative	55	21.34	PSL (10 mg/day)	Good	[5]
3	83	F	Exudative effusion (increased neutrophils)	Negative	N.A.	19.25	PSL (20 mg/day)	Good	[6]
4	76	M	Exudative effusion (increased eosinophils)	Negative	79	9.35	PSL (20 mg/day)	Good	[7]
5	76	M	Effusion (angioimmunoblastic T-cell lymphoma)	Positive	N.A.	3.38	PSL (20 mg/day) + THP-CHOP	Death	[8]
Our case	74	F	Exudative effusion (increased neutrophils)	Positive	44	6.7	Steroid pulse^b^ + PSL (15 mg/day)	Good	

^a^half steroid pulse – intravenous hydrocortisone 500 mg/day for 3 days, ^b^steroid pulse – intravenous methylprednisolone 1000 mg/day for 3 days, *CRP* C-reactive protein, *ESR* erythrocyte sedimentation rate, *F* female, *M* male, *N.A.* not available, *PSL* prednisolone, *Ref* reference, *THP-CHOP* adriamycin+cyclophosphamide+Pinorubin (pirarubicin hydrochloride)+vincristine+prednisolone

Several reports suggested that serum IL-6 and VEGF are increased in RS3PE syndrome [2, 10–12], suggesting that these cytokines may be associated with symptoms of RS3PE syndrome. In our patient, serum IL-6 and VEGF were also markedly elevated when pleural and pericardial effusions were increased. After steroid therapy, levels of serum IL-6 and VEGF decreased when pleural and pericardial effusions disappeared. IL-6 is an important cytokine that induces VEGF production [13]. VEGF induces vascular endothelial cell proliferation, enhances angiogenesis, and accelerates vascular permeability [13]. Elevated VEGF and IL-6 may be associated with the cause of pleural and pericardial effusions in idiopathic RS3PE syndrome.

In general, idiopathic RS3PE syndrome is considered to be a benign disorder; however, patients with RS3PE syndrome may develop life-threatening respiratory and circulatory problems due to pleuritis and endocarditis. Therefore, physicians should be aware of these complications when diagnosing RS3PE syndrome and determining treatment.

## Conclusions

Our findings suggest that pleuritis and endocarditis are rare complications in idiopathic RS3PE syndrome. These complications may be associated with increased serum VEGF and IL-6.
